# Integrating a Postpartum Contraception Intervention in the Maternal and Child Health Care System of China: A Randomized Clinical Trial

**DOI:** 10.1001/jamanetworkopen.2024.50635

**Published:** 2024-12-13

**Authors:** AnXin Yin, XiaoYan Zhou, Xu Qian, Lei Zhang, XiuRui Wang, HuiBin Yang, YuHan Song, LongMei Jin, Mu Li, Hong Jiang

**Affiliations:** 1 School of Public Health, National Health Commission Key Laboratory of Health Technology Assessment, Fudan University, Shanghai, China; 2 Minhang District Maternal and Child Health Hospital, Shanghai, China; 3 School of Public Health, The University of Sydney, Sydney, Australia

## Abstract

**Importance:**

The interpregnancy intervals are suboptimal worldwide, and women lack access to high-quality postpartum contraceptive services in China.

**Objective:**

To evaluate the effectiveness of integrating postpartum contraceptive interventions into the existing maternal and child health care (MCH) system.

**Design, Setting, and Participants:**

This cluster randomized trial randomly allocated 13 communities in Minhang District in Shanghai, China, to the intervention or the control group. Data were collected between September 2020 and February 2023. Eligible participants included women who registered their pregnancy in community health centers and were followed up to 1 year post partum. Data analysis was conducted from April 2023 to May 2024.

**Interventions:**

Participants in the intervention group received postpartum contraception consultations, online educational videos, a 45-minute class, health prescriptions, and educational pamphlets at 5 stages in alignment with the MCH system of China. The control group received routine MCH services.

**Main Outcomes and Measures:**

The primary outcome was incidence of unintended pregnancy within 1 year after childbirth. The secondary outcomes included knowledge level of postpartum contraception, utilization rate of reliable long-acting reversible contraception (LARC), and incidence of induced abortion.

**Results:**

A total of 1279 participants (698 in intervention from 7 clusters and 581 in control from 6 clusters) were recruited, and 995 (521 older than 30 years [40.7%]; 539 intervention [54.2%] and 456 control [45.8%]) completed the trial. The incidence of unintended pregnancy, LARC utilization, and induced abortion within 1 year after childbirth was 1.5% (8 participants), 3.2% (17 participants), and 0.6% (3 participants), respectively, in the intervention group, and 3.9% (18 participants), 1.1% (5 participants), and 2.0% (9 participants), respectively, in the control group. Participants in the intervention group were significantly less likely to experience unintended pregnancy (adjusted odds ratio [aOR], 0.33; 95% CI, 0.16-0.70), had a higher rate of LARC utilization (aOR, 2.47; 95% CI, 1.02-5.98), and a lower rate of induced abortion due to unintended pregnancy (aOR, 0.30; 95% CI, 0.09-0.99). The postpartum contraception knowledge level in the intervention group was substantially higher than the control group (β = 24.20; 95% CI, 20.92-27.47).

**Conclusions and Relevance:**

This cluster randomized clinical trial demonstrated the designed postpartum contraceptive interventions were effective in reducing unintended pregnancy and induced abortion and increasing postpartum contraception knowledge and LARC utilization among women at 1-year post partum.

**Trial Registration:**

Chictr.org.cn Identifier: ChiCTR2000034603

## Key Points

**Question:**

Is integrating postpartum contraceptive interventions into the existing maternal and child health care system effective at reducing the incidence of unintended pregnancy and promoting women’s reproductive health?

**Findings:**

In this cluster randomized clinical trial including 13 communities and 1279 participants, the intervention significantly reduced the unintended pregnancy rate from 3.9% to 1.5%.

**Meaning:**

These findings suggest a potential implementation strategy for postpartum contraceptive intervention to reduce unintended pregnancy, support optimal interpregnancy intervals, and in turn, improve universal reproductive health care coverage.

## Teaser

This cluster randomized clinical trial assesses the effects of a contraception intervention on the incidence of unintended pregnancy at 1 year post partum among women in the maternal and child health care (MCH) system of China.

## Introduction

Access to family planning is vital for the health and well-being of both mothers and children. It plays a key role in achieving the sustainable development goal of ensuring universal access to sexual and reproductive health.^1^ Postpartum family planning is defined as preventing closely spaced or unintended pregnancies within the first 12 months following childbirth, allowing women to make informed decisions about the timing and number of children they have.^1,2^ The World Health Organization (WHO) recommends a minimum birth spacing of 24 months to reduce the risk of negative outcomes for both mother and child.^3^

The time interval between a live birth and a subsequent gestation, known as interpregnancy interval (IPI), has significant impacts on maternal and child health.^4^ Shorter IPIs are associated with an increased risk of adverse maternal and neonatal outcomes.^5^ These include low birth weight, preterm birth, small for gestational age, and perinatal death.^6,7,8,9,10,11^ Women with shorter IPIs also faced an increased risk of obesity and gestational diabetes,^12,13^ antenatal and postpartum hemorrhage, premature rupture of membranes, and other pregnancy and childbirth complications.^3,14,15^ However, World Health Organization guidance on IPIs was not widely followed, especially in low-income and middle-income countries.^16^

In China, the incidence of postpartum unintended pregnancy is more than 5% within the first year after childbirth and more than 10% within 2 years.^17,18,19^ Improper use of contraceptives and limited access to postpartum contraception education contributed to this situation.^17,18,19^ In the first year after childbirth more than 70% of pregnancies were unintentional,^20^ and more than 75% of postpartum unintentional pregnancies ended in abortion.^18^ There were misunderstandings of postpartum contraception, leading to a lack of effective contraceptive practices among postpartum couples.^21,22,23^ Most Chinese women chose condoms over more reliable, long-acting reversible contraception (LARC).^23^ The low LARC utilization rate (<10%) contributed to higher risks of postpartum unintended pregnancy and induced abortion.^18,19,24,25^ Chinese postnatal health care staff lacked the knowledge and capability to provide contraceptive services,^23,26^ and there was a small-magnitude association of maternal and child health care (MCH) with postpartum contraception services.

In this cluster randomized clinical trial in Shanghai, China, we aimed to evaluate the effectiveness of integrating postpartum contraceptive interventions into the existing MCH system; specifically, the efficacy of this intervention in reducing the incidence of unintended pregnancy and induced abortion, as well as increasing the postpartum contraception knowledge level and LARC utilization rate within 1 year post partum were assessed.

## Methods

### Study Design

This study was a cluster randomized trial involving all 13 communities in Minhang District in Shanghai, China, to evaluate the effectiveness of postpartum contraception intervention in reducing unintended pregnancy and induced abortion and increasing the postpartum contraception knowledge level and LARC utilization rate. Each community was randomly allocated to the intervention or the control group, and data were collected between September 2020 and February 2023. Details of the study design and protocol for this postpartum contraception intervention research were previously described and published,^27^ and the research protocol is provided in Supplement 1. This study followed the Consolidated Standards of Reporting Trials Extension (CONSORT Extension) reporting guideline.^28^ The study has been approved by the ethics committee of the Shanghai Minhang District Maternal and Child Health Hospital.^27^ All participants provided written informed consent.

### Study Population

All the 13 communities in Minhang District in Shanghai, China, were included as clusters of the study and were randomly assigned to either the intervention group or control group. We recruited eligible women who registered their pregnancy in community health centers (CHCs) of the 13 communities between September 2020 and April 2021. Categorized by the community of residence, participants were enrolled into either the intervention group or the control group at baseline and subsequently followed up to 1 year post partum. Participant eligibility criteria were with the ability to read and understand Chinese, planning to deliver and live in Minhang District until 1 year after childbirth, consenting to be followed up, and having a WeChat account to complete the online questionnaires.^27^ Exclusion criteria included abortion or stillbirth during the index pregnancy, baby in special care nursery, and loss to follow-up.^27^ Participants without sexual intercourse during 1 year postpartum or who divorced were excluded for all outcomes except for knowledge level of postpartum contraception.

### Randomization and Blinding

Communities were assigned to the intervention or the control group by a computer-generated random number. In this research, each of the 13 communities was regarded as an individual cluster. Due to the nature and design of the study, blinding at the intervention level was not feasible. While intervention clinicians and participants were not obscured from group assignments, blinding procedure was implemented during the data analysis stage to minimize potential biases.

### Intervention and Control

Training on postpartum contraception intervention services was provided for relevant health staff in our study and details were provided in published protocol.^27^ In addition to the routine prenatal and postnatal health care package, we delivered interventions to participants in the intervention group at 5 stages in alignment with the MCH system of China. (1) At pregnancy registration, health staff in CHCs offered the first consultation, emphasized the importance of postpartum contraception, and provided educational videos. (2) At the second and third trimesters, obstetricians and obstetric nurses in the hospital provided a 45-minute postpartum contraception class with face-to-face explanations of postpartum contraception knowledge. (3) From childbirth to discharge from hospital, obstetricians and obstetric nurses in maternity wards provided education and advice including contraceptive counselling and a health educational prescription, as well as recommended exclusive breastfeeding and LARC to women without contraindications. (4) At the postpartum home visits, the community health staff assisted participants to choose suitable contraceptive measures according to their conditions and provided educational pamphlets containing knowledge on postpartum contraception. (5) At the 42-day postpartum examination in the childbirth hospital, obstetricians provided face-to-face counselling, an individualized health prescription based on participants’ chosen contraceptive method, and information about the access to free contraceptives and designated hospitals for the placement of subcutaneous implants or intrauterine devices.^27^ Due to the COVID-19 pandemic and lockdown, we provided the 42-day postpartum intervention via telephone, and the knowledge assessment at this stage was also cancelled. The summary of interventions at different stages is provided in the published protocol.^27^

No additional training on postpartum contraception was conducted for service clinicians in the control group. Participants assigned to the control group received routine MCH services and conventional education on postpartum contraception during postnatal home visits and 42-day postpartum health examinations (Supplement 1).^27^ In routine health care package, community health staff provided basic advice on contraception during postnatal home visits, and obstetricians would briefly recommend possible contraceptive methods during the 42-day examination.

### Data Collection

All participants were followed up from their first trimesters to 1 year post partum (Supplement 1). During pregnancy registration, health care staff in CHCs collected participants’ baseline information face to face using a self-administered questionnaire, including age, residence, education, parity, history of miscarriage, history of unintended pregnancy, and a short contraceptive knowledge test. At postpartum home visits, participants were asked to complete a questionnaire face to face, including questions about their pregnancy outcomes, plans for future pregnancy and postpartum contraception, and a postpartum contraceptive knowledge test.

The baseline contraceptive knowledge test included 12 questions (maximum score 12) to gather information on whether the participants were aware of any postpartum contraceptive methods or had previously used these methods. The contraceptive knowledge test at postpartum home visits was more comprehensive (maximum score 100), aiming to evaluate the intervention effect on participants’ postpartum contraceptive knowledge. It included questions about participants’ understanding of highly effective contraceptive methods and LARCs, appropriate contraceptive options for women with different postpartum conditions, the criteria for the lactational amenorrhea method, and the health impact of pregnancy within the first year post partum on mothers and newborns. At 6 months and 1 year after childbirth, health staff in CHCs collected all participants’ information on their choices and implementation of contraceptive methods as well as conception and abortion conditions via telephone interviews.

### Outcomes

The primary outcome was the incidence of unintended pregnancy within 1 year after childbirth. The secondary outcomes involved the knowledge level of postpartum contraception within 1 week after birth during the postpartum home visit. Additionally, secondary outcomes included the utilization rate of LARC and the incidence of induced abortion due to unintended pregnancy within 1 year after childbirth. Data for these outcomes was collected via telephone interviews conducted by health staff in CHCs at 1 year post partum. Sample size and statistical power calculation for each outcome are described in the published protocol.^27^

### Statistical Analysis

All major analyses were conducted using the intention-to-treat approach, and sensitivity analyses were conducted using the per-protocol approach. We employed multiple imputations to address missing data and generated an intention-to-treat dataset including all participants. We summarized the baseline characteristics of each group using descriptive analysis and assessed the differences between groups using the χ^2^ test or the Student *t* test. We compared primary and secondary outcomes between the intervention and the control group using χ^2^ tests for the rate of unintended pregnancy, LARC utilization, and induced abortion and using the Student *t* test for postpartum contraception knowledge level. The effects of the intervention were analyzed using the linear mixed-effects model for the continuous outcome with the link function (identity link) or the generalized linear mixed model for the binary outcomes with the logistic model (logit link). We conducted both unadjusted and adjusted model analyses and incorporated a random intercept for each community in the model, accounting for the random effects of community clustering. We used SPSS version 25.0 (IBM Corporation) and R version 4.1.3 (R Project for Statistical Computing) to conduct statistical tests. All statistical analyses were 2-sided, and *P* < .05 was defined as statistical significance. Analysis occurred from April 2023 to May 2024.

## Results

### Baseline Characteristics

**Figure 1.**
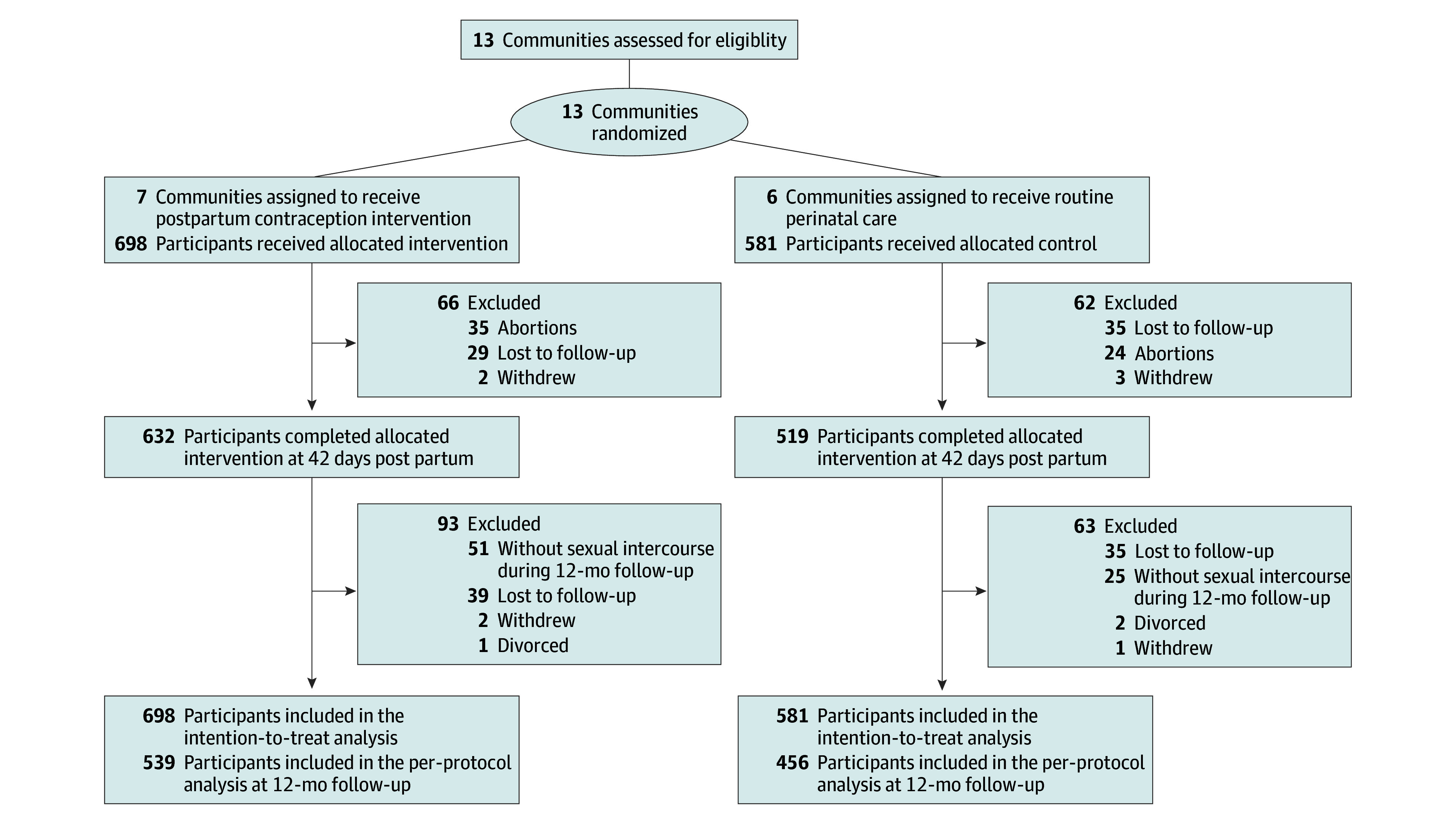
Participant Flow Diagram

Of the 1279 participants (698 in the intervention group from 7 clusters; 581 in the control group from 6 clusters) who were recruited and randomized, a total of 539 pregnant women in the intervention group and 456 pregnant women in the control group completed the trial (Figure 1). All participants self-reported as Han Chinese, while 521 participants (40.7%) were older than 30 years, 946 (74.0%) had an educational level of college and above, and 804 (62.9%) were primiparous. The mean (SD) score of participants’ contraceptive knowledge level at baseline was 8.35 (2.63) out of 12. The randomization produced the intervention and control groups with similar baseline characteristics, including age, residence, education, parity, history of miscarriage, history of unintended pregnancy, and baseline contraceptive knowledge level (Table and eTable 1 in Supplement 2). Participants with missing data had no significant differences in group allocation and baseline characteristics except for lower educational level (eTable 2 in Supplement 2).

**Table.** Baseline Characteristics of the Participants by Study Group

Characteristic	Individuals, No. (%)		
	Intervention (n = 698)	Control (n = 581)	Total (N = 1279)
Age, y			
≤30	414 (59.3)	344 (59.2)	758 (59.3)
>30	284 (40.7)	237 (40.8)	521 (40.7)
Permanent residence registration			
Local residence	296 (42.4)	261 (44.9)	557 (43.5)
Nonlocal residence	402 (57.6)	320 (55.1)	722 (56.5)
Education level			
Junior school and below	92 (13.2)	66 (11.4)	158 (12.4)
Senior high school or vocational school	104 (14.9)	71 (12.2)	175 (13.7)
College and above	502 (71.9)	444 (76.4)	946 (74.0)
Parity			
Primiparous	439 (62.9)	365 (62.8)	804 (62.9)
Multiparous	259 (37.1)	216 (37.2)	475 (37.1)
History of miscarriage			
No	446 (63.9)	356 (61.3)	802 (62.7)
Yes	252 (36.1)	225 (38.7)	477 (37.3)
History of unintended pregnancy			
No	480 (68.8)	412 (70.9)	892 (69.7)
Yes	218 (31.2)	169 (29.1)	387 (30.3)
Baseline contraceptive knowledge level score, mean [SD]^a^	8.47 (2.60)	8.20 (2.66)	8.35 (2.63)

^a^ The baseline contraceptive knowledge level was assessed on a scale ranging from 0 to 12.

### Primary Outcome

The incidence of unintended pregnancy within 1 year after childbirth was 1.5% (8 participants) in the intervention group and 3.9% (18 participants) in the control group (eTable 3 in Supplement 2). Compared with the control group, the incidence of unintended pregnancy was significantly lower in the intervention group (χ^2^_1_ = 5.889; *P* = .02).

**Figure 2.**
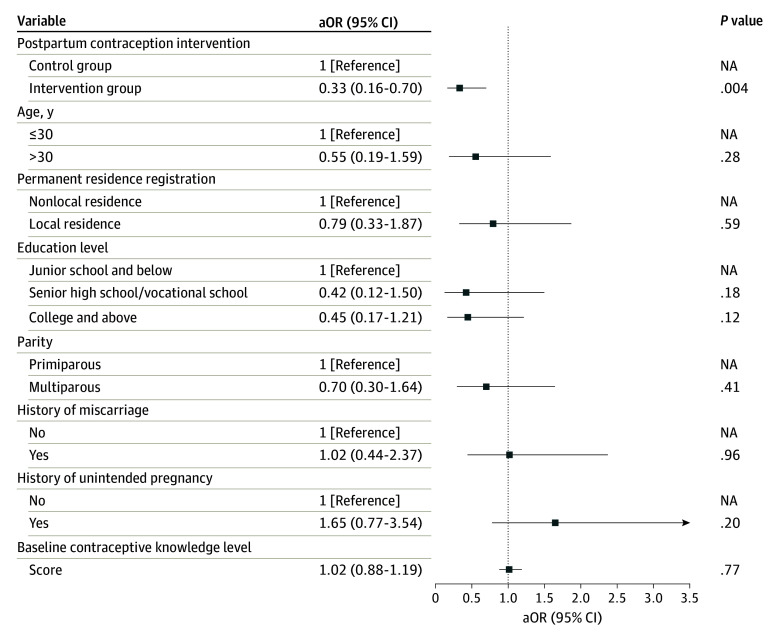
Generalized Linear Mixed Model for Unintended Pregnancy aOR indicates adjusted odds ratio; NA, not applicable.

The generalized linear mixed model adjusted for age, residence, education, occupation, and parity showed that participants in the postpartum intervention group were significantly less likely to experience unintended pregnancy within 1 year after childbirth than those in the control group (adjusted odds ratio [aOR], 0.33; 95% CI, 0.16-0.70; *P* = .004) (Figure 2). Sensitivity analysis using the per-protocol population also demonstrated that the intervention decreased the risk of unintended pregnancy within 1 year after childbirth (eFigure 1 in Supplement 2). Results of the unadjusted model are shown in eTable 4 in Supplement 2.

### Secondary Outcomes

The utilization rate of LARC at 1 year post partum was 3.2% (17 participants) in the intervention group and 1.1% (5 participants) in the control group, and the rate of induced abortion due to unintended pregnancy within 1 year postpartum was 0.6% (3 participants) in the intervention group and 2.0% (9 participants) in the control group (eTable 3 in Supplement 2). The mean (SD) score of postpartum contraception knowledge level was 61.09 (18.62) in the intervention group and 36.34 (17.92) in the control group (eTable 5 in Supplement 2). Compared with the control group, women in the intervention group had higher postpartum contraception knowledge level (*t* = 20.707; *P* < .001), were significantly more likely to use LARC (χ^2^_1_ = 4.836; *P* = .03), and were less likely to have induced abortion due to unintended pregnancy (χ^2^_1_ = 4.163; *P* = .04).

**Figure 3.**
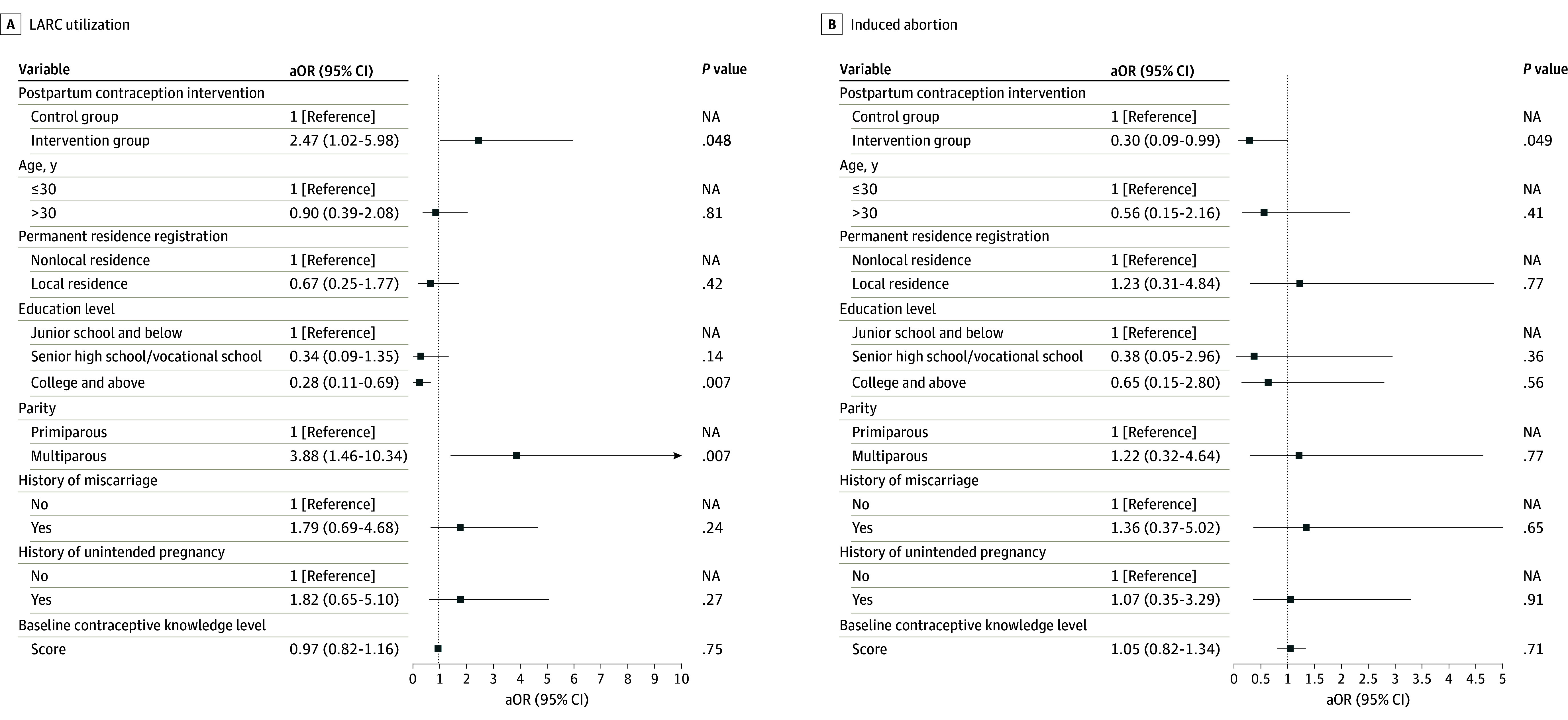
Generalized Linear Mixed Model for Long-Acting Reversible Contraception (LARC) Utilization and Induced Abortion aOR indicates adjusted odds ratio; NA, not applicable.

**Figure 4.**
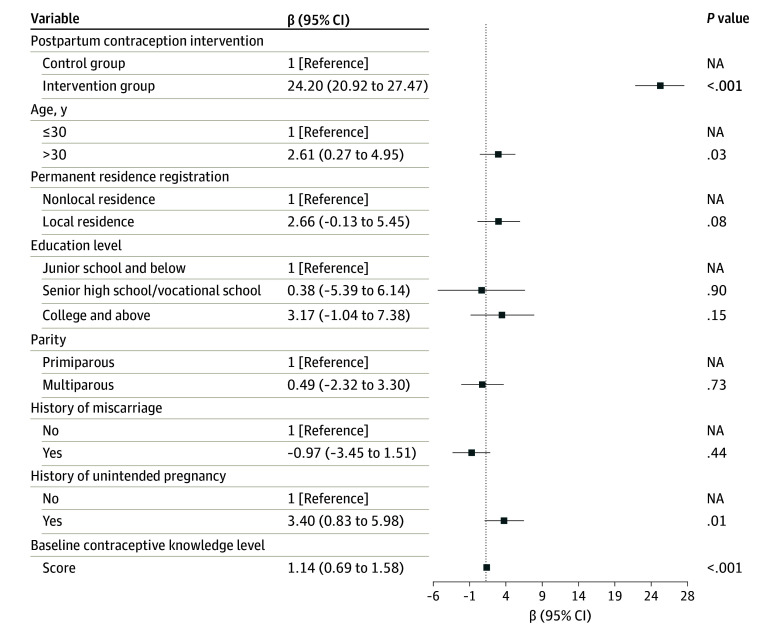
Linear Mixed-Effects Model for Postpartum Contraception Knowledge Level

In adjusted analyses, results showed that participants in the intervention group had a significantly higher rate of LARC utilization (aOR, 2.47; 95% CI, 1.02-5.98; *P* = .048), and a significantly lower rate of induced abortion due to unintended pregnancy (aOR, 0.30; 95% CI, 0.09-0.99; *P* = .049) within 1 year after childbirth compared with the control group (Figure 3). In addition, the linear mixed-effects model analysis indicated that the postpartum contraception knowledge level was increased substantially by the intervention (β = 24.20; 95% CI, 20.92-27.47; *P* < .001) (Figure 4). Sensitivity analysis based on the per-protocol population demonstrated similar results (eFigure 2 and eFigure 3 in Supplement 2). Results of the unadjusted model are shown in eTable 5 in Supplement 2.

## Discussion

In this cluster randomized clinical trial, we found that women in the postpartum contraceptive intervention group had a significantly lower risk of unintended pregnancy and induced abortion within 1 year post partum and had a higher postpartum contraception knowledge level and LARC utilization rate compared with women in the control group. These results indicate that in China, integrating postpartum contraception intervention into the existing MCH system could be an effective approach to improve postpartum women’s contraception practices and reduce unintended pregnancies.

A Chinese national survey^18^ including 19 939 postpartum women reported the incidence of unintended pregnancy was 5.3% within 1 year after childbirth. In our study, the incidence of unintended pregnancy in the control group was 3.9% at endline, likely due to more developed economy and education in Shanghai. Our intervention further reduced the unintended pregnancy rate to 1.5% and decreased abortion rates from 2.0% to 0.6%. These results were consistent with several previous Chinese studies.^29,30,31^ A stepped-wedge cluster randomized trial in Nepal^32^ also indicated that counselling in both predischarge and postdischarge periods reduced the unmet need for postpartum contraception and offered help for postpartum women at risk of unintended pregnancy. In addition, our findings were in agreement with literature that supported continuous interventions for women at multiple stages across the continuum of MCH system rather than counselling alone to improve postpartum contraception practices.^33,34^ Our results suggested that a continuum of interventions integrated into the routine MCH system, covering both the prenatal and postpartum periods, would help to reduce the incidence of unintended pregnancies and abortions; this is likely because well-designed interventions have increased women’s understanding and prioritization of contraception and enhanced their postpartum contraceptive practices.

The study found women in the intervention group had a significantly higher LARC utilization rate post partum compared with the control group. This result aligned with some previous trials^35,36^ that showed educational tools about LARC efficiently increased the uptake of postpartum LARC. However, the overall LARC utilization rate in both the intervention group (3.2%) and control group (1.1%) was low in this study, which may be due to the traditional beliefs that LARC would harm maternal health, as well as insufficient contraceptive knowledge among Chinese women.^37,38^ Furthermore, due to the COVID-19 pandemic and lockdown, the intervention approach at 42 days post partum was changed from in-person to telephone, which also potentially affected and explained the low rate of LARC uptake in our study. These reasons were also consistent with Burapasikarin et al,^35^ who found that accessibility problems and uncertainty about LARC were the leading reasons for not using LARC. It is recommended to use other research methods, such as qualitative studies, to understand the detailed reasons for low utilization of LARC in this population.

The intervention group showed a significant increase in postpartum contraception knowledge level compared with the control group, indicating that our intervention effectively enhanced women’s knowledge of postpartum contraception. Further exploration is needed to translate the knowledge enhancement into improved postpartum contraceptive practices.

The strengths of our research lie in the well-designed and feasible intervention strategies and integration with existing MCH systems. The cluster randomized clinical trial design including 13 communities and the substantial sample size also contributed to the robustness of the study results.

The practical significance of the study is ensuring appropriate IPIs and improving women’s reproductive health. A recent systematic review and meta-analysis of 16 studies with 14 289 participants^39^ showed no significant associations of current interventions (such as counselling, education, and reminders) with women’s contraceptive use, repeat pregnancies, and induced abortions during 1 year post partum, emphasizing the necessity for further research to formulate more feasible strategies. Our research findings indicate integrating the postpartum contraceptive intervention into the existing Chinese MCH system can effectively reduce the risk of unintended pregnancies. Women had a high incidence of unintended pregnancy post partum, with rates reaching up to 44% during the first postpartum year.^35^ Unintended pregnancy was found to be significantly associated with adverse maternal and infant outcomes.^40^ Our study suggested a potential implementation strategy for postpartum contraceptive intervention to improve universal reproductive health care coverage.

### Limitations

There are several potential limitations. The intervention clinicians and participants could not be blinded to the group allocation due to the nature and design of the study intervention. However, we minimized potential bias by prespecifying outcomes and analyses and blinding statisticians during data analyses. Although the exclusion rates were similar between the 2 groups, differences in education levels between participants retained and those lost to follow-up could introduce potential bias in the results. Considering the low LARC utilization in the study, taking a mixed-methods approach in future studies, with a larger sample size and incorporating qualitative research, is warranted. In addition, we designed the intervention model based on the current Chinese MCH system, so the intervention model may not be suitable to be directly generalized to other countries. Nevertheless, we believe that this intervention model can contribute to the evidence of the effectiveness of postpartum contraception interventions in promoting women’s reproductive health.

## Conclusions

The findings of this cluster randomized clinical trial indicate the designed postpartum contraceptive interventions are effective in reducing the incidence of unintended pregnancy and induced abortion, as well as increasing the postpartum contraception knowledge level and LARC utilization among women at 1 year post partum. Our results provide crucial evidence supporting the potential benefits of integrating contraceptive interventions into the existing MCH system to promote women’s reproductive health. Further research with a longer follow-up period is necessary to evaluate the long-term effect of the postpartum contraceptive interventions.

## Notes

Supplement 1.
Trial Protocol

Supplement 2. **eTable 1.** Differences of Baseline Characteristics of the Participants by Study Group **eTable 2.** Characteristics of the Participants by Follow-Up Completion **eTable 3.** Univariate Analysis for the Effect of Postpartum Contraception Intervention (Per-Protocol Dataset) **eFigure 1.** Generalized Linear Mixed Model for Unintended Pregnancy (Per-Protocol Dataset) **eTable 4.** Unadjusted Model Analysis for the Effect of Postpartum Contraception Intervention **eTable 5.** Univariate Analysis for the Effect of Postpartum Contraception Intervention on Contraception Knowledge Level (Per-Protocol Dataset) **eFigure 2.** Generalized Linear Mixed Model for LARC Utilization and Induced Abortion (Per-Protocol Dataset) **eFigure 3.** Linear Mixed Effects Model for Postpartum Contraception Knowledge Level (Per-Protocol Dataset)

Supplement 3.
Data Sharing Statement
