# Supplementary material for: Integrating a Postpartum Contraception Intervention in the Maternal and Child Health Care System of China: A Randomized Clinical Trial
Source: JAMA Netw Open. 2024 Dec 13;7(12):e2450635. doi: 10.1001/jamanetworkopen.2024.50635 (PMC11645651; doi:10.1001/jamanetworkopen.2024.50635)
Supplement: Supplement 1. — Trial Protocol [file jamanetwopen-e2450635-s001.pdf]

Integrating contraceptive services into existing perinatal care: protocol for a community-based cluster randomised controlled trial in Shanghai, China

ABSTRACT

Introduction: Postpartum contraception is essential to preventing unintended pregnancies and short interpregnancy intervals. The first year after childbirth is a critical period with a high risk of unintended pregnancy and induced abortion. However, the postpartum contraceptive services are weak in China's existing maternal and child health care system. We propose to evaluate the effects of integrating postpartum contraceptive services into the existing perinatal care system via a cluster randomised controlled trial.

Methods and analysis: This cluster randomised controlled trial involves all 13 communities of Minhang District, Shanghai, China. Communities will be randomly allocated, seven in the intervention group and six in the control group. One thousand and three hundred women, 100 women in each community, will be recruited in the study. Women assigned to the intervention group will receive postpartum contraceptive education and counselling during pregnancy, childbirth hospitalization, postpartum home visits, and the 42-day postpartum clinic check-up. Women in the control group will receive routine antenatal and postpartum care. Participants will be recruited in the first trimester during pregnancy and followed up to one year postpartum. The primary outcome is the incidence of unintended pregnancy within one year after childbirth.

Ethics and dissemination: The trial received ethical approval from the Ethics Committee of Shanghai Minhang District Maternal and Child Health Care Hospital (#[2020]KS-02, #[2020]KS-05, #[2020]KS-05-EX). Results will be published in academic journals and disseminated in multiple formats for the health professionals and the public.

Trial registration number: Chinese Clinical Trial Registry (ChiCTR2000034603)

Keywords: Postpartum contraception, Postpartum women, Postpartum family planning, Perinatal care, Cluster randomised controlled trial

INTRODUCTION

Postpartum contraception plays an important role in preventing unintended pregnancies and short intervals between pregnancies[1]. The World Health Organization (WHO) recommends birth spacing should be at least 24 months to reduce adverse pregnancy and birth outcomes and to improve child health[2].

Interpregnancy intervals (IPIs), defined as the time interval between live birth and the beginning of the next pregnancy, are closely related to birth outcomes[1]. Short IPIs are associated with an increased risk of several adverse birth outcomes, such as preterm birth, low birth weight (LBW), small for gestational age (SGA) and perinatal death[3-6]. IPIs are also related to maternal outcomes. A systematic review showed that among women who had undergone a trial of labour and ended in low transverse caesarean section, short IPIs (<16 months) increased the risk of uterine rupture[2]. Women with short IPIs were more likely to experience chronic diseases, such as obesity and gestational diabetes[7 8]. Moreover, women with short IPIs had a significantly increased risk of maternal mortality, antenatal bleeding, premature rupture of membranes and anaemia, which seriously affected women's physical and mental health as well as family and social harmony[9].

In China, the rate of induced abortion due to unintended pregnancies has constantly increased, 50.3% were postpartum women[10]. A study in 2018 showed that the first year after childbirth was a period with a high risk of unintended pregnancy and induced abortion[11 12]. At least 70% of pregnancies in the first year after childbirth were unintentional[13]. The number of induced abortions within one year after childbirth accounted for 10.76% of the total number of induced abortions[14]. More than half (56.1%) of the women who had induced abortions within one year postpartum did not take any contraception[14]. Accumulated evidence has shown there were various misunderstandings in postpartum contraception, which led to not taking contraception or not adopting efficient contraceptive methods among couples[15]. Some women believed that they would not get pregnant after childbirth before menstruation resuming[16]. In addition, with the concern about the side effects on lactation and infant growth, a growing number of women chose short-term methods, such as condoms rather than more reliable long-acting reversible contraception (LARC), which increased the risk of unintended pregnancy[17 18].

In China, women register their pregnancy and have their first antenatal examinations in community health centres (CHCs) within three months. After registration at CHC, they will have regular antenatal examinations and antenatal classes provided by obstetricians and obstetric nurses in local hospitals before childbirth. After childbirth, women will stay in maternity wards for at least 24 hours for observation and receive maternal and neonatal health care. Then, at 3-7 days and 14-28 days after hospital discharge, health staff in CHCs will conduct postpartum home visits to check mothers' and newborns' health status as well as provide health care advice. At 42 days after childbirth, women and their newborns will return to the childbirth hospitals for check-ups by obstetricians. Currently, women only have chances of receiving simple postpartum contraceptive education after childbirth, including two times of postpartum home visits and the 42-day postpartum check-up. However, obstetric staff providing postpartum care in China were found to lack contraceptive knowledge and service capability[19]. For example, they mainly recommended condoms as the postpartum contraceptive choice rather than more effective methods such as LARC[19]. Due to insufficient obstetric personnel capacity and other constraints, most women cannot get timely contraceptive services within

one year after childbirth. Furthermore, approximately half of the women resumed sexual intercourse within six weeks after childbirth, which indicated family planning services provided after childbirth might be too late to prevent unintended pregnancy[20 21]. However, the link between maternal health care and the family planning services is weak in China, and access to quality postpartum contraceptive services is often difficult.

With China's new policy encouraging couples to have three children[22], postpartum contraception is even more critical in promoting the physical and mental recovery of postpartum women, maintaining reasonable birth spacing, improving early childhood development and enhancing family and social harmony. Therefore, we propose a cluster randomised controlled trial to examine whether postpartum contraceptive interventions integrated into the existing perinatal care system would reduce unintended pregnancy among women within one year after childbirth in Shanghai, China.

Study objectives and hypotheses

This study aims to assess the effectiveness of integrating postpartum conception services into the existing perinatal care system in Shanghai, China, to prevent unintended pregnancy among women within one year after childbirth. We hypothesise that the intervention will:

1) reduce the rate of unintended pregnancy within one year after childbirth.
2) improve women's knowledge of postpartum contraception.
3) improve the utilization of long-acting reversible contraception within one year after childbirth.
4) improve the postpartum contraceptive service capacity of obstetric medical personnel in Minhang District of Shanghai.

The study will provide evidence for establishing a service model of postpartum contraception integrated into the perinatal care system to meet the postpartum contraception service needs in Shanghai, China.

METHODS AND ANALYSIS

Study design

Using the cluster randomised control trial study design, all of the 13 communities in Minhang District will be involved as the research sites in Shanghai, China. Each community will be randomly allocated to either the intervention or the control group (see Fig. 1 for research flow). Ethical approval to conduct this trial has been granted by the Ethics Committee of Shanghai Minhang District Maternal and Child Health Care Hospital (#[2020]KS-02, #[2020]KS-05, #[2020]KS-05-EX). The research is registered with Chinese Clinical Trial Registry (#ChiCTR2000034603). The first participant was recruited on 21 September 2020. We anticipate completing our data collection by April 2023.

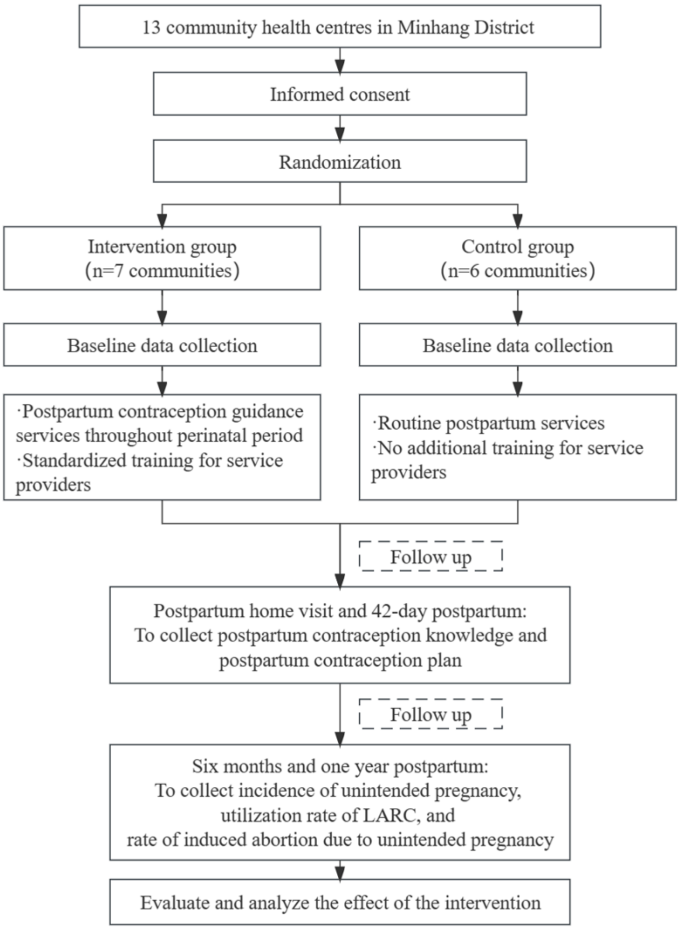

Fig. 1 Research flow chart

Participants and recruitment

This study will be conducted in Minhang District, in the central region of Shanghai. It has 13 communities, with a total population of 2,653,489 in 2021. The region's GDP was slightly lower than the average level of administrative districts of Shanghai.

All 13 communities in the district will be included in this trial and be randomly assigned to the intervention and control groups. In China, women register their pregnancy in community health centres (CHCs) within three months after pregnancy. They will receive perinatal care in the hospitals they choose to give birth and CHCs until 42 days postpartum. Women who register their pregnancy in CHCs and are eligible for the study will be invited to participate in the study by the health staff in CHCs. Each participant will be required to sign a written informed consent.

Inclusion criteria

Pregnant women

1)With the ability to read and understand Chinese

2)With the plan to live in Minhang District from the pregnancy registration to one year after childbirth

3)With the plan to give birth in a childbirth hospital in Minhang District

4)Consent to be followed up until one year after childbirth

5)With the WeChat account through which the online questionnaire survey can be fulfilled

Exclusion criteria

1)Miscarriage

2)Stillbirth

3)Baby in special care nursery

4)Loss to follow-up after discharge from hospital following childbirth

Randomization

Random allocation to the intervention and the control group will be determined by a computer-generated random number. We take one community as a cluster in the randomization and the total number of the clusters is 13. After randomization, there will be seven clusters in the intervention group and six clusters in the control group[23].

Blinding

Due to the nature of the intervention, service providers and users will not be blinded to the group allocation. The statistician will be blinded for the group allocation of participants during data analysis.

Intervention group

Training for service providers

Face-to-face training on postpartum contraception services was provided for community health staff responsible for pregnancy registration in CHCs and postpartum home visits, and obstetricians and obstetric nurses responsible for antenatal classes, maternity ward care and 42-day postpartum examination in hospitals.

The training material contents were designed by the research group with experts in gynaecology and obstetrics, maternal and child health care and family planning. International, national and local guidelines and service norms, including *Chinese experts' consensus on the clinical use of female contraceptive methods*[13], *The technical guide of long-acting reversible contraceptives for those post-abortion and postpartum women*[24] and *Ensuring human rights in the provision of contraceptive information and services: guidance and recommendations*[25] were used. The experts reviewed and streamlined the resources to establish the essential contents of the training into modules. The core training modules consist of five parts, including informed consent of project services, optimal time and duration of postpartum contraception, basic principles and methods of common contraception, recommendation of LARC methods, and need-based individualised contraceptive counselling. The service providers were also trained in communication skills and the standard process of filling in questionnaires.

Before and after the training, quizzes were carried out to evaluate the effect of the training. Practical training such as scenario-based role-play and counselling

practice in the simulated situation was held. There were two theoretical and four practical training sessions, and one reinforcement session. Each session lasted for one day. Service providers will not participate in the intervention until they complete all the training sessions and pass the training tests.

Intervention for service users

We will carry out interventions at five stages in alignment with the current perinatal care system of China.

1. Participants will be recruited after giving informed consent at pregnancy registration. Then health staff in CHCs will offer the first consultation for the intervention group, emphasise the importance of postpartum contraception, and provide educational videos via the WeChat platform. The videos could be watched repeatedly at convenient times and contain the introduction of the necessity of postpartum contraception and various contraceptive methods such as the lactational amenorrhoea method (LAM) and LARC.
2. At the second and third trimesters, the intervention aims to stimulate interest in a postpartum contraceptive plan. Participants in the intervention group will take a 45-minute postpartum contraception class given by obstetricians and obstetric nurses in the hospital antenatal classes, which include appropriate methods, common misunderstandings, and recommendations for postpartum contraception. Specifically, when participants have their antenatal examinations, obstetricians will make an appointment for them to attend this postpartum contraception class. Obstetricians and obstetric nurses will use the same multimedia materials and video prepared by our research team and provide explanations face to face during the contraception class.
3. From childbirth to discharge from hospital, participants will be provided with education and advice by obstetricians and obstetric nurses in maternity wards. The key messages will include the ovulation resumption time of different breastfeeding methods, optimal IPIs, available contraceptive measures after childbirth, and LAM criteria. Obstetricians will conduct contraceptive counselling and recommend exclusive breastfeeding and LARC to women without contraindications. The husbands will be encouraged to participate in the counselling with their wives, and support postpartum contraception. Additionally, couples will get a health educational prescription after the counselling.
4. At the postpartum home visits (3-7 days and 14-28 days after discharge), the community health staff will help participants to choose suitable contraceptive measures according to their conditions. Further, participants will receive educational pamphlets containing knowledge on postpartum contraception.
5. At the 42-day postpartum check-up in the childbirth hospital, obstetricians will provide participants with face-to-face counselling and an individualised health prescription based on the contraceptive method they choose. To promote the implementation of postpartum contraception, participants will be informed about the access to free contraceptives and designated hospitals for the placement of subcutaneous implants or intrauterine devices (IUD). The interventions at different stages are summarized in Table 1.

Table 1. Summary of the intervention

Stage	Intervention contents	Service providers	Approac h
Pregnancy	Project introduction, counselling, and	Health staff in	Face-to-f

registration	educational videos to inform the importance of postpartum contraception	CHCs	ace counselling<br>Online video
Second and third trimesters	Class in hospitals containing key knowledge of postpartum contraception to stimulate interest in a postpartum contraceptive plan	Obstetricians and obstetric nurses	Class in hospital
Childbirth hospitalization	Counselling and health prescriptions to facilitate making the postpartum contraceptive plan	Obstetricians and obstetric nurses in maternity wards	Face-to-face counselling<br>Prescription
Postpartum home visit	Counselling and educational pamphlets offering contraceptive knowledge and advice to assist in completing the postpartum contraceptive plan	Health staff in CHCs	Face-to-face counselling<br>Pamphlet
42-day postpartum check-up	Counselling, health prescriptions, and information about the access to postpartum services to promote the utilization of postpartum contraception	Obstetricians in childbirth hospitals	Face-to-face counselling<br>Prescription

*CHCs, community health centres

Control group

Postpartum contraceptive service providers in the control group will not receive additional training on postpartum contraception. Women in the control group will receive routine perinatal care and regular postpartum contraceptive education at postpartum home visits and 42-day postpartum health check-ups. In routine perinatal health care, the community health staff will remind women to consider postpartum contraception, ask about their postpartum contraceptive plan, and provide brief recommendations on postpartum contraception (mainly condoms) during the postpartum home visit. At 42-day postpartum health check-ups in the childbirth hospital, obstetricians will remind women to choose and implement appropriate postpartum contraception methods based on their conditions, but without specific consultation and instructions.

Data collection

All participants will be followed up from the first trimester antenatal to 42 days postnatal period. They will be asked to complete the questionnaires at five time points:1) pregnancy registration; 2) first postpartum home visit at 3-7 days; 3) 42 days after childbirth; 4) six months after childbirth; 5) one year after childbirth.

At baseline data

In China, pregnant women register their pregnancy in CHCs to establish pregnancy records and have their first antenatal examinations. At the pregnancy registration, health staff in CHCs will ask participants to complete a self-administered questionnaire via scanning the QR code or clicking the link of the questionnaire website to collect their baseline information. The baseline information will include

participants' demographic characteristics (age, residence, educational level, occupation), obstetric history, contraceptive knowledge and their need for postpartum contraception services.

At postpartum home visit

The health staff in the CHCs will conduct the first postpartum home visit for women in 3-7 days after hospital discharge. Participants in both groups will complete an online questionnaire, which includes questions about their delivery outcomes, a postpartum contraceptive knowledge test, and their plans for the next pregnancy and postpartum contraception. Participants in the intervention group will fill in their contraception plan after the postpartum contraception counselling. Health staff in CHCs will ensure participants' informed decision-making by providing them with information on the advantages, disadvantages, and applicable conditions of various contraceptive methods.

On 42-day postpartum check-up

At 42 days after childbirth, postpartum women will return to the childbirth hospital for obstetric examinations. Women in the intervention group will be asked to complete an online questionnaire. Through the questionnaire, obstetricians of the childbirth hospital will collect their lactation and sexual behaviour information as well as their postpartum contraception condition, and confirm whether they receive the postpartum contraceptive intervention services of each stage. For those who haven't initiate any contraception, obstetricians will collect their plan for postpartum contraception after the counselling. In addition, another postpartum contraceptive knowledge test will be included in the questionnaire to collect participants' knowledge of postpartum contraception.

At half-year and one year postpartum

At half a year and one year after childbirth, information on participants' selections of contraceptive methods, their utilization frequency and satisfaction of the chosen contraception, conception condition and abortion experience will be collected via telephone interviews by health staff in CHCs.

Primary outcome

Incidence of unintended pregnancy

The primary outcome of this study is the incidence of unintended pregnancy within one year after childbirth, which will be collected at one year postpartum via telephone interviews. Health staff in CHCs will ask participants whether they fall pregnant after childbirth or not, and whether it is planned or unintended. The information on frequency, time, and outcome of the pregnancy within one year after childbirth will also be collected. The incidence of unintended pregnancy will be calculated and compared between the intervention group and the control group.

Secondary outcomes

Utilization rate of LARC

At one year postpartum, participants will be asked whether they take postpartum contraception and the method they choose via telephone interviews. The health staff

in CHCs will collect information on contraceptive methods participants use through a multiple-choice question with five response selections: common methods such as condoms and in vitro ejaculation, LARC such as IUD and subcutaneous implant, short-acting oral contraceptives, emergency contraception pills (ECPs), and sterilization. Participants will also be asked about their utilization frequency of the chosen contraception. The utilization rate of LARC within one year after childbirth will be calculated based on the above information.

Rate of induced abortion due to unintended pregnancy

Postpartum women will be asked whether they fall pregnant via telephone interviews at one year after childbirth. For pregnant women, health staff in CHCs will ask whether the pregnancy is planned or unintended, and the outcomes, including induced abortion and continued gestation. The rate of induced abortion due to unintended pregnancy within one year after childbirth will be calculated.

Knowledge of postpartum contraception

Participants' knowledge of postpartum contraception will be assessed via online questionnaires at pregnancy registration, postpartum home visit and 42 days after childbirth respectively. At the baseline survey during pregnancy registration, a list of contraceptive methods will be displayed and women will be asked to choose the methods that they have heard of or used before. At the postpartum home visit, a knowledge test including questions about LARC, recommended contraceptive methods with different lactation plans, LAM criteria and adverse impacts of short IPIs on mothers and children will be conducted. At 42 days after childbirth, participants in the intervention group will complete another knowledge test, and they will be asked about recommended contraceptive methods with different lactation plans, LAM criteria, optimal IPIs, the recommended time of resuming sexual behaviour and taking postpartum contraception after childbirth.

Sample size and statistical power calculation

Sample size calculation for primary outcome

There are a total of 13 communities in Minhang District, Shanghai. The sample size was calculated based on the primary outcome of the incidence of unintended pregnancy within one year postpartum. We used PASS 15.0 to calculate the sample size. The estimated incidence of unintended pregnancy within one year postpartum was 10% in the control group[26], the expected difference between the intervention group and the control group was 6%[27], and the intracluster correlation coefficient (ICC) was 0.01[28]. A sample size of 1040 pregnant women (80 in each CHC) will be needed at 0.05 significance level and 80% statistical power. Given the 20% anticipated rate of loss to follow-up from recruitment to one year after childbirth, a total of 1300 women (100 in each community health service centre) will be required.

Calculation of statistical power for secondary outcomes

The power calculations for secondary outcomes include the utilization rate of LARC, induced abortion rate of unintended pregnancy, and knowledge of postpartum contraception.

Utilization rate of LARC

The estimated LARC utilization rate in the control group was 25% and the expected Odds Ratio was 1.6 between the two groups[29]. Given the calculated sample size of 80 per cluster based on the primary outcome, the statistical power will be 97.5% for the utilization rate of LARC at 0.05 significance level.

Rate of induced abortion due to unintended pregnancy

The estimated induced abortion rate within one year postpartum in the control group was 10%, and the expected Odds Ratio was 0.4 between the two groups[30 31]. Given the calculated sample size of 80 per cluster based on the primary outcome, the statistical power will be 80.0% for the rate of induced abortion due to unintended pregnancy at 0.05 significance level.

Knowledge of postpartum contraception

The estimated accuracy rate of postpartum contraception questions in the control group was 65%, and the expected Odds Ratio was 1.2 between the two groups[32]. Given the calculated sample size of 80 per cluster based on the primary outcome, the statistical power will be 93.6% for the postpartum contraception knowledge at 0.05 significance level.

Data management

A unique identification number will be assigned to women once they agree to participate in the study at the pregnancy registration. Personal information will not be identifiable.

All information will be collected through the online questionnaire and stored on the questionnaire platform. The data will be secured with an account and password, and access to information will be limited to research team members. Researchers of Fudan University will be responsible for data security.

Data analysis

Descriptive analysis will be performed to examine all variables. For categorical variables, frequency and percentages will be reported, while mean ± SD will be reported for continuous variables.

The baseline characteristics of the intervention and the control group such as age and pregnancy history will be compared. All outcomes will be compared between the intervention and the control group. The difference between the two groups will be assessed by conducting parametric tests (t-test and ANOVA) or non-parametric tests (Wilcoxon, Kruskal-Wallis and Friedman tests) for continuous variables. The Chi-square test will be used for categorical variables. The effect of the intervention will be evaluated by generalised linear mixed model (GLMM). The evaluation of the study will be based on the “intention to treat” analysis. SPSS software (version 25.0, IBM Corporation) and R software (version 4.1.3) will be used to conduct the statistical tests.

Process evaluation

The process evaluation and quality control will be conducted by the research group in Minhang District Maternal and Child Health Care Hospital and the participating CHCs. We will conduct pre-surveys to ensure the validity and reliability of the research questionnaires by checking ambiguities and semantic expressions. Service providers will be trained according to the protocol to ensure the feasibility and quality of the intervention. To reduce invalid questionnaires, we will set logic checks for online questionnaires so that questionnaires with unreasonable responses or incomplete items will not be submitted. The designated project managers and investigators will act as quality controllers, and will be responsible for monitoring the recruitment process on the sites and making records. During the intervention, both service providers and participants in the intervention group will be asked to sign a confirmation form after each face-to-face intervention at pregnancy registration, postpartum hospitalization, postpartum home visits, and 42-day postpartum check-up as an implementation process recording. Key components of the intervention at each stage will be listed on the form, and the participants will confirm whether they received the intervention by signing at the end of the form. The quality controllers will check these forms routinely to ensure the implementation is consistent with the plan. In addition, based on the records and periodical summary of site supervision, we will hold regular meetings with experts and staff of CHCs and childbirth hospitals every two months to solve the existing problems and ensure intervention protocol compliance.

Patient and Public Involvement statement

During the study design, we conducted a formative study on the current postpartum contraceptive services from women to understand their service needs. We also sought opinions and suggestions on intervention strategy from obstetricians and obstetric nurses in maternal and child health institutions to ensure the feasibility of the intervention. Furthermore, we conducted a pilot survey among ten pregnant women during the first trimester and ten postpartum women, and improved the questionnaires based on their feedback. All pilot participants have confirmed that the questionnaires were easy to understand without ambiguity or obscurity. Postpartum women suggested adding a satisfaction survey for intervention services to monitor and improve the intervention process. In addition, participants of the pilot survey proposed to add a question collecting the specific contraceptive method recommended by service providers during the counselling.

DISCUSSION

A growing body of research has shown that contraceptive services are effective interventions to improve maternal and infant health outcomes[33-36]. The postpartum period is critical to adopt appropriate and effective contraceptive methods to reduce unintended pregnancies[36]. To address the unmet service needs on postpartum contraception and tackle the challenges, in this proposed study, we will integrate postpartum contraceptive services into the current perinatal care system from the first trimester to 42 days postpartum. We hypothesise that the intervention will reduce the rate of unintended pregnancy and increase the utilization of LARC within one year after childbirth, as well as improve women’s knowledge of postpartum contraception.

If the intervention is proven effective, this service model will be up-scaled in all maternal and child health care institutions, including CHCs and hospitals in Minhang District of Shanghai, China. We expect that our findings will promote postpartum contraception, support women in making informed contraception decisions, and improve postpartum contraception services.

This trial will develop and evaluate a postpartum contraception intervention for women based on their needs. The intervention strategies will help to reduce the rate of unintended pregnancy in the first year after childbirth. It will potentially contribute to postpartum women's physical and mental rehabilitation, maintaining a reasonable birth interval and achieving family and social harmony.

There are several anticipated limitations of this study. First, the intervention model was designed based on the current perinatal care system in urban areas of Shanghai, China, so it may not be applicable to rural areas. Second, participant retention may be challenging as the follow-up will be about 20 months, i.e., from pregnancy to one year postpartum. To minimise the rate of loss to follow-up, we will implement the intervention in the existing maternal care system, tapping in the usual care to deliver the intervention contents.

Ethics and dissemination

The study has been approved by the Ethics Committee of Shanghai Minhang District Maternal and Child Health Hospital (#[2020]KS-02, #[2020]KS-05, #[2020]KS-05-EX). The research is registered with Chinese Clinical Trial Registry (#ChiCTR2000034603). All participants are required to provide written informed consent. All research activities will be carried out in accordance with relevant guidelines and regulations. The data will be confidential after the study completion. The data generated in this study will be available from the corresponding author on reasonable request. Results of the study will be published in academic journals and be disseminated in research seminars and other appropriate formats for the professionals and the public.

REFERENCES

1. Sridhar A, Salcedo J. Optimizing maternal and neonatal outcomes with postpartum contraception: impact on breastfeeding and birth spacing. *Matern Health Neonatol Perinatol* 2017;**3**:1. doi: 10.1186/s40748-016-0040-y

2. World Health Organization. Report of a WHO technical consultation on birth spacing: Geneva, Switzerland 13-15 June 2005. Geneva, Switzerland: World Health Organization 2007.

3. Winikoff B. The Effects of Birth Spacing on Child and Maternal Health. *Population Council* 1983;**14**:231-45.

4. Rousso D, Panidis D, Gkoutzioulis F, *et al.* Effect of the interval between pregnancies on the health of mother and child. *European Journal of Obstetrics & Gynecology and Reproductive Biology* 2002;**105**(1):4-6. doi: 10.1016/s0301-2115(02)00077-5

5. King JC. The risk of maternal nutritional depletion and poor outcomes increases in early or closely spaced pregnancies. *J Nutr* 2003;**133**(5 Suppl 2):1732S-36S. doi: 10.1093/jn/133.5.1732S

6. Conde-Agudelo A, Rosas-Bermudez A, Kafury-Goeta AC. Birth spacing and risk of adverse perinatal outcomes: a meta-analysis. *JAMA* 2006;**295**(15):1809-23. doi: 10.1001/jama.295.15.1809

7. Hanley GE, Hutcheon JA, Kinniburgh BA, *et al.* Interpregnancy Interval and Adverse Pregnancy Outcomes: An Analysis of Successive Pregnancies. *Obstet Gynecol* 2017;**129**(3):408-15. doi: 10.1097/AOG.0000000000001891

8. Davis EM, Babineau DC, Wang X, *et al.* Short inter-pregnancy intervals, parity, excessive pregnancy weight gain and risk of maternal obesity. *Matern Child Health J* 2014;**18**(3):554-62. doi: 10.1007/s10995-013-1272-3

9. ACOG Practice Bulletin No. 121: Long-acting reversible contraception: Implants and intrauterine devices. *Obstet Gynecol* 2011;**118**(1):184-96. doi: 10.1097/AOG.0b013e318227f05e

10. Hou ZH, Gu XY, Wu SC. The Technical Guide of Long-acting Reversible Contraceptives for those Post-abortion and Postpartum Women [in Chinese]. *J Int Reprod Health/Fam Plan* 2013;**32**(04):267-68+89.

11. Huang YM, Kang JZ, Hu XY, *et al.* Analysis of unintended pregnancy and its influencing factors among postpartum women in Shanghai [in Chinese]. *Reproduction & Contraception* 2008;**5**(28).

12. Gu XY, Che Y. Insight of the big data about artificial abortion and contraception in family planning work after the birth control policy was eased in China [in Chinese]. *Chinese Journal of Practical Gynecology and Obstetrics* 2018;**34**(01):46-51.

13. Cheng LN, Di W, Ding Y, *et al.* Chinese expert consensus on clinical application of female contraceptive methods [in Chinese]. *Shanghai Medical Journal* 2018;**41**(11):641-55.

14. Yang YP, Liu YL, Guo PP, *et al.* Investigation on induced abortion of 344 cases of unintended pregnancy within one year after childbirth [in Chinese]. *The Journal of Practical Medicine* 2016;**32**(04):659-61.

15. Chen WL, Lv CS, Liu XY. Misunderstandings and countermeasures of postpartum contraception [in Chinese]. *Chinese Journal Of Family Planning & Gynecotokology* 2019;**11**(1):15-17. doi: 10.3969/j.issn.1674-4020.2019.01.05

16. Speroff L, Mishell DR, Jr. The postpartum visit: it's time for a change in order to optimally initiate contraception. *Contraception* 2008;**78**(2):90-8. doi: 10.1016/j.contraception.2008.04.005

17. Wang CT. Trends in contraceptive use and determinants of choice in China: 1980–2010. *Contraception* 2012;**85**(6):570-79. doi: 10.1016/j.contraception.2011.10.014

18. Liberty A, Yee K, Darney BG, *et al.* Coverage of immediate postpartum long-acting reversible contraception has improved birth intervals for at-risk populations. *Am J Obstet Gynecol* 2020;**222**(4S):S886 e1-S86 e9. doi: 10.1016/j.ajog.2019.11.1282

19. Jin LM, Cui W, Yang HB, *et al.* Survey on contraceptive knowledge and service ability among postpartum visitors in Shanghai Minhang district [in Chinese]. *Chin J Gen Pract* 2019;**18**(8):742-45. doi: 10.3760/cma.j.issn.1671-7368.2019.08.007

20. Woranitat W TS. Sexual Function during the Postpartum Period. *J Med Assoc Thai* 2007;**90**:1744–8.

21. Connolly A, Thorp J, Pahel L. Effects of pregnancy and childbirth on postpartum sexual function: a longitudinal prospective study. *Int Urogynecol J Pelvic Floor Dysfunct* 2005;**16**(4):263-7. doi: 10.1007/s00192-005-1293-6

22. State Council of the People's Republic of China: Decision on Optimizing the Birth Policy and Promoting the Long-term and Balanced Development of Population

[online]. 2021. http://www.gov.cn/zhengce/2021-07/20/content_5626190.htm (accessed 5 Feb 2022).
23. He Y, Zhang N, Wang J, *et al.* Evaluation of two intervention models on contraceptive attitudes and behaviors among nulliparous women in Shanghai, China: a clustered randomized controlled trial. *Reprod Health* 2017;**14**(1):73. doi: 10.1186/s12978-017-0331-4
24. Hou ZH, Wu SC, Gu XY. The Technical Guide of Long-acting Reversible Contraceptives for those Post-abortion and Postpartum Women [in Chinese]. *J Int Reprod Health/Fam Plan* 2013(4):267-68,89.
25. Rodriguez. MI, Kismodi. E, Cottingham. J, *et al.* Ensuring Human Rights in the Provision of Contraceptive Information and Services: Guidance and Recommendations. Geneva, Switzerland: World Health Organization 2014.
26. Xiao H, Qin CP, Yan H, *et al.* Prevalence and reasons for unexpected pregnancy during postpartum period among married women in Enshi, China [in Chinese]. *Chin J Dis Control Prev* 2014;**18**(9):876-79.
27. Zhang Y, Liu JH, Yang L. Evaluation of the effect of contraception education in late pregnancy and postpartum on postpartum women's unintended pregnancy [in Chinese]. *Maternal and Child Health Care of China* 2021;**36**(10):2389-93. doi: 10.19829/j.zgfybj.issn.1001-4411.2021.10.064
28. Adams G, Gulliford MC, Ukoumunne OC, *et al.* Patterns of intra-cluster correlation from primary care research to inform study design and analysis. *Journal of clinical epidemiology* 2004;**57**(8):785-94. doi: 10.1016/j.jclinepi.2003.12.013
29. Tran NT, Seuc A, Coulibaly A, *et al.* Post-partum family planning in Burkina Faso (Yam Daabo): a two group, multi-intervention, single-blinded, cluster-randomised controlled trial. *The Lancet Global Health* 2019;**7**(8):e1109-e17. doi: 10.1016/S2214-109X(19)30202-5
30. Xi W, Liu Y, Jian CX, *et al.* Influence of individual contraceptive intervention on postpartum women [in Chinese]. *Chinese Journal of Reproduction and Contraception* 2022;**42**(9):924-32. doi: 10.3760/cma.j.cn101441-20210728-00326
31. He L. The effect of family planning guidance on postpartum contraceptive compliance rate and success rate [in Chinese]. *Contemporary Medicine Forum* 2018;**16**(4):70-72. doi: 10.3969/j.issn.2095-7629.2018.04.048
32. Yu XP, Qian FB, Shen Y, *et al.* Influence of diversified health education led by WeChat on postpartum women 's contraceptive cognition level and contraceptive behavior [in Chinese]. *International Journal of Nursing* 2021;**40**(22):4096-100. doi: 10.3760/cma.j.cn221370-20200329-01153
33. Rice LW, Espey E, Fenner DE, *et al.* Universal access to contraception: women, families, and communities benefit. *American Journal of Obstetrics and Gynecology* 2020;**222**(2):150.e1-50.e5. doi: 10.1016/j.ajog.2019.09.014
34. Ganatra B, Faundes A. Role of birth spacing, family planning services, safe abortion services and post-abortion care in reducing maternal mortality. *Best Practice & Research Clinical Obstetrics & Gynaecology* 2016;**36**:145-55. doi: 10.1016/j.bpobgyn.2016.07.008
35. Makins A, Cameron S. Post pregnancy contraception. *Best Pract Res Clin Obstet Gynaecol* 2020;**66**:41-54. doi: 10.1016/j.bpobgyn.2020.01.004
36. Gaffield ME, Egan S, Temmerman M. It's about time: WHO and partners release programming strategies for postpartum family planning. *Global health, science and practice* 2014;**2**(1):4-9. doi: 10.9745/ghsp-d-13-00156
