# Supplementary material for: Integrating a Postpartum Contraception Intervention in the Maternal and Child Health Care System of China: A Randomized Clinical Trial
Source: JAMA Netw Open. 2024 Dec 13;7(12):e2450635. doi: 10.1001/jamanetworkopen.2024.50635 (PMC11645651; doi:10.1001/jamanetworkopen.2024.50635)
Supplement: Supplement 2. — eTable 1. Differences of Baseline Characteristics of the Participants by Study Group eTable 2. Characteristics of the Participants by Follow-Up Completion eTable 3. Univariate Analysis for the Effect of Postpartum Contraception Intervention (Per-Protocol Dataset) eFigure 1. Generalized Linear Mixed Model for Unintended Pregnancy (Per-Protocol Dataset) eTable 4. Unadjusted Model Analysis for the Effect of Postpartum Contraception Intervention eTable 5. Univariate Analysis for the Effect of Postpartum Contraception Intervention on Contraception Knowledge Level (Per-Protocol Dataset) eFigure 2. Generalized Linear Mixed Model for LARC Utilization and Induced Abortion (Per-Protocol Dataset) eFigure 3. Linear Mixed Effects Model for Postpartum Contraception Knowledge Level (Per-Protocol Dataset) [file jamanetwopen-e2450635-s002.pdf]

Supplemental Online Content

Yin A, Zhou X, Qian X, et al. Integrating a postpartum contraception intervention in the maternal and childcare system of china: a randomized clinical trial. *JAMA Netw. Open*. 2024;7(12):e2450635. doi:10.1001/jamanetworkopen.2024.50635

eTable 1. Differences of Baseline Characteristics of the Participants by Study Group
eTable 2. Characteristics of the Participants by Follow-Up Completion
eTable 3. Univariate Analysis for the Effect of Postpartum Contraception Intervention (Per-Protocol Dataset)
eFigure 1. Generalized Linear Mixed Model for Unintended Pregnancy (Per-Protocol Dataset)
eTable 4. Unadjusted Model Analysis for the Effect of Postpartum Contraception Intervention
eTable 5. Univariate Analysis for the Effect of Postpartum Contraception Intervention on Contraception Knowledge Level (Per-Protocol Dataset)
eFigure 2. Generalized Linear Mixed Model for LARC Utilization and Induced Abortion (Per-Protocol Dataset)
eFigure 3. Linear Mixed Effects Model for Postpartum Contraception Knowledge Level (Per-Protocol Dataset)

This supplemental material has been provided by the authors to give readers additional information about their work.

© 2024 Yin AX et al. *JAMA Network Open.*

eTable 1. Differences of Baseline Characteristics of the Participants by Study Group

Characteristic	Individuals, No. (%)				
	Intervention (n=698)	Control (n=581)	Total (n=1279)	χ^2 or t	P value
Age					
⩽30	414 (59.3)	344 (59.2)	758 (59.3)	0.001	.97
>30	284 (40.7)	237 (40.8)	521 (40.7)		
Permanent residence registration					
Local residence	296 (42.4)	261 (44.9)	557 (43.5)	0.816	.37
Non-local residence	402 (57.6)	320 (55.1)	722 (56.5)		
Education level					
Junior school and below	92 (13.2)	66 (11.4)	158 (12.4)	3.383	.18
Senior high school/Vocational school	104 (14.9)	71 (12.2)	175 (13.7)		
College and above	502 (71.9)	444 (76.4)	946 (74)		
Parity					
Primiparous	439 (62.9)	365 (62.8)	804 (62.9)	0.816	.37
Multiparous	259 (37.1)	216 (37.2)	475 (37.1)		
History of miscarriage					
No	446 (63.9)	356 (61.3)	802 (62.7)	0.933	.33
Yes	252 (36.1)	225 (38.7)	477 (37.3)		
History of unintended pregnancy					
No	480 (68.8)	412 (70.9)	892 (69.7)	0.691	.41
Yes	218 (31.2)	169 (29.1)	387 (30.3)		
Baseline contraceptive knowledge level[a]	8.47±2.60	8.20±2.66	8.35±2.63	1.816	.07

[a] The baseline contraceptive knowledge level was assessed on a scale ranging from 0 to 12.

© 2024 Yin AX et al. *JAMA Network Open.*

eTable 2. Characteristics of the Participants by Follow-Up Completion

Characteristic	Individuals, No. (%) Complete follow-up (n=995)	Incomplete follow-up (n=284)	χ^2 or t	*P* value
Group				
Control	456 (45.8)	159 (56.0)	0.294	.59
Intervention	539 (54.2)	125 (44.0)		
Age				
⩽30	586 (58.9)	172 (60.6)	0.255	.61
>30	409 (41.1)	112 (39.4)		
Permanent residence registration				
Local residence	435 (43.7)	122 (43.0)	0.052	.82
Non-local residence	560 (56.3)	162 (57.0)		
Education level				
Junior school and below	106 (10.7)	52 (18.3)	14.986	.001
Senior high school/Vocational school	130 (13.1)	45 (15.8)		
College and above	759 (76.3)	187 (65.8)		
Parity				
Primiparous	623 (62.6)	181 (63.7)	0.119	.73
Multiparous	372 (37.4)	103 (36.3)		
History of miscarriage				
No	624 (62.7)	178 (62.7)	0.000	.99
Yes	371 (37.3)	106 (37.3)		
History of unintended pregnancy				
No	687 (69.0)	205 (72.2)	1.031	.31
Yes	308 (31.0)	79 (27.8)		
Baseline contraceptive knowledge level[a]	8.41±2.59	8.13±2.78	1.579	.12

[a] The baseline contraceptive knowledge level was assessed on a scale ranging from 0 to 12.

© 2024 Yin AX et al. *JAMA Network Open.*

eTable 3. Univariate Analysis for the Effect of Postpartum Contraception Intervention (Per-Protocol Dataset)

	Individuals, No. (%)				
**Outcome**	**Intervention (n=539)**	**Control (n=456)**	**Total (n=995)**	χ^2	***P* value**
Unintended pregnancy					
Yes	8 (1.5)	18 (3.9)	26 (2.6)	5.889	.015
No	531 (98.5)	438 (96.1)	969 (97.4)		
Utilization of LARC					
Yes	17 (3.2)	5 (1.1)	22 (2.2)	4.836	.028
No	522 (96.8)	451 (98.9)	973 (97.8)		
Induced abortion due to unintended pregnancy					
Yes	3 (0.6)	9 (2.0)	12 (1.2)	4.163	.041
No	536 (99.4)	447 (98.0)	983 (98.8)		

Abbreviations: LARC, long-acting reversible contraception.

eFigure 1. Generalized Linear Mixed Model for Unintended Pregnancy (Per-Protocol Dataset)

Variable	Adjusted odds ratio (95% CI)	P value
Postpartum contraception intervention		
Control group	1.00	-
Intervention group	0.35 (0.15-0.82)	.02
Age		
≤30	1.00	-
>30	0.58 (0.23-1.47)	.25
Permanent residence registration		
Non-local residence	1.00	-
Local residence	0.86 (0.38-1.97)	.73
Education level		
Junior school and below	1.00	-
Senior high school/Vocational school	0.47 (0.11-2.07)	.32
College and above	0.51 (0.17-1.54)	.23
Parity		
Primiparous	1.00	-
Multiparous	0.94 (0.37-2.41)	.90
History of miscarriage		
No	1.00	-
Yes	1.24 (0.50-3.05)	.64
History of unintended pregnancy		
No	1.00	-
Yes	1.61 (0.67-3.92)	.29
Baseline contraceptive knowledge level		
Score	1.04 (0.89-1.22)	.62

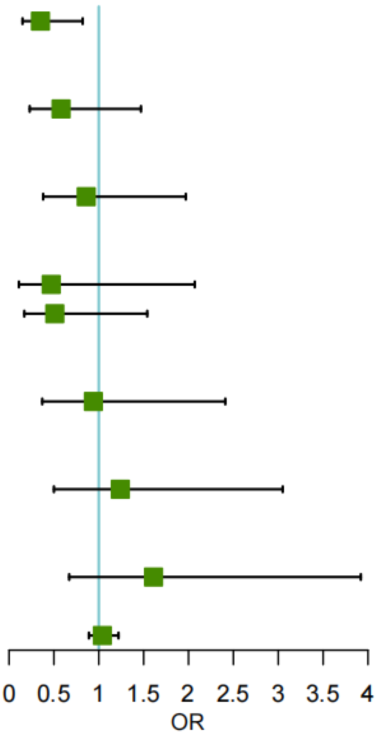

© 2024 Yin AX et al. *JAMA Network Open.*

eTable 4. Unadjusted Model Analysis for the Effect of Postpartum Contraception Intervention

Outcome	Unadjusted odds ratio or β[a]	95% CI	*P* value
**Intention-To-Treat Dataset**			
Unintended pregnancy	0.35	0.17-0.73	.006
Utilization of LARC	2.29	0.90-5.84	.09
Induced abortion due to unintended pregnancy	0.31	0.09-1.00	.05
Postpartum contraception knowledge level	24.36	20.90-27.81	<.001
**Per-Protocol Dataset**			
Unintended pregnancy	0.36	0.15-0.82	.02
Utilization of LARC	2.70	0.98-7.41	.05
Induced abortion due to unintended pregnancy	0.27	0.07-1.00	.05
Postpartum contraception knowledge level	24.70	20.47-28.92	<.001

Abbreviations: LARC, long-acting reversible contraception.

[a] Odds ratio for Unintended pregnancy, Utilization of LARC, Induced abortion due to unintended pregnancy; β for Postpartum contraception knowledge level.

© 2024 Yin AX et al. *JAMA Network Open.*

eTable 5. Univariate Analysis for the Effect of Postpartum Contraception Intervention on Contraception Knowledge Level (Per-Protocol Dataset)

	Individuals, No. (%)				
**Outcome**	**Intervention (n=512)**	**Control (n=433)**	**Total (n=945)**	**t**	***P* value**
Postpartum contraception knowledge level					
Score (out of 100)	61.09±18.62	36.34±17.92	49.75±22.07	20.707	<.001

© 2024 Yin AX et al. *JAMA Network Open.*

eFigure 2. Generalized Linear Mixed Model for LARC Utilization and Induced Abortion (Per-Protocol Dataset)

Variable	LARC utilization (Per-Protocol Dataset)		Induced abortion (Per-Protocol Dataset)	
	Adjusted odds ratio (95% CI)	P value	Adjusted odds ratio (95% CI)	P value
Postpartum contraception intervention				
Control group	1.00	-	1.00	-
Intervention group	2.80 (0.99-7.86)	.05	0.27 (0.07-1.03)	.06
Age				
≤30	1.00	-	1.00	-
>30	0.69 (0.27-1.78)	.45	0.71 (0.20-2.58)	.60
Permanent residence registration				
Non-local residence	1.00	-	1.00	-
Local residence	0.64 (0.24-1.72)	.38	1.37 (0.42-4.47)	.60
Education level				
Junior school and below	1.00	-	1.00	-
Senior high school/Vocational school	0.41 (0.12-1.42)	.16	0.41 (0.04-4.69)	.47
College and above	0.30 (0.11-0.83)	.02	0.69 (0.13-3.69)	.66
Parity				
Primiparous	1.00	-	1.00	-
Multiparous	6.82 (1.82-25.53)	.004	1.76 (0.46-6.75)	.41
History of miscarriage				
No	1.00	-	1.00	-
Yes	1.96 (0.67-5.70)	.22	1.62 (0.44-5.96)	.47
History of unintended pregnancy				
No	1.00	-	1.00	-
Yes	1.78 (0.70-4.50)	.23	0.85 (0.22-3.30)	.82
Baseline contraceptive knowledge level				
Score	0.98 (0.84-1.15)	.81	1.05 (0.84-1.33)	.65

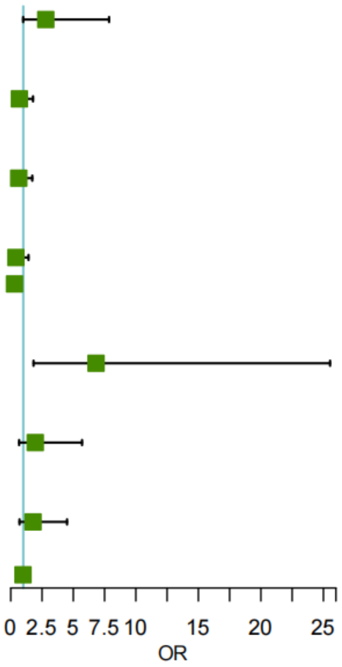

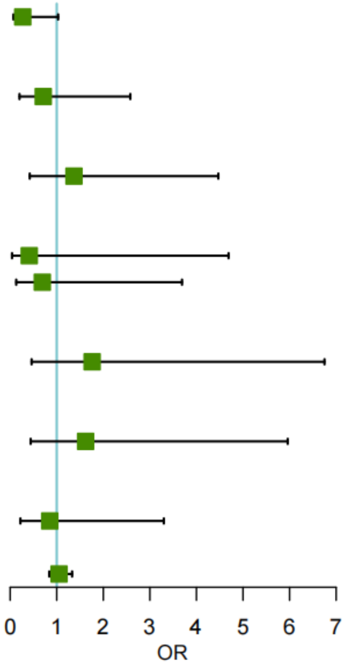

LARC: long-acting reversible contraception

© 2024 Yin AX et al. *JAMA Network Open.*

eFigure 3. Linear Mixed Effects Model for Postpartum Contraception Knowledge Level (Per-Protocol Dataset)

Variable	β (95% CI)	P value
Postpartum contraception intervention		
Control group	-	-
Intervention group	24.64 (20.65-28.63)	<.001
Age		
≤30	-	-
>30	2.83 (0.32-5.35)	.03
Permanent residence registration		
Non-local residence	-	-
Local residence	3.10 (0.77-5.44)	.009
Education level		
Junior school and below	-	-
Senior high school/Vocational school	-0.38 (-4.99-4.23)	.87
College and above	3.26 (-0.65-7.16)	.10
Parity		
Primiparous	-	-
Multiparous	0.28 (-2.48-3.05)	.84
History of miscarriage		
No	-	-
Yes	-0.48 (-3.20-2.25)	.73
History of unintended pregnancy		
No	-	-
Yes	3.00 (0.26-5.73)	.03
Baseline contraceptive knowledge level		
Score	1.04 (0.58-1.49)	<.001

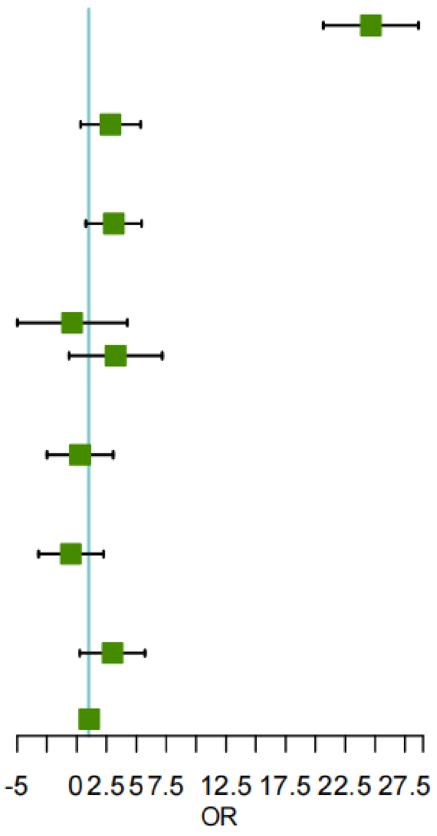

© 2024 Yin AX et al. *JAMA Network Open.*
