# Supplementary material for: Integrating a Postpartum Contraception Intervention in the Maternal and Child Health Care System of China: A Randomized Clinical Trial
Source: JAMA Netw Open. 2024 Dec 13;7(12):e2450635. doi: 10.1001/jamanetworkopen.2024.50635 (PMC11645651; doi:10.1001/jamanetworkopen.2024.50635)
Supplement: Supplement 3. — Data Sharing Statement [file jamanetwopen-e2450635-s003.pdf]

Data Sharing Statement

Yin. Integrating a Postpartum Contraception Intervention in the Maternal and Child Health Care System of China. *JAMA Netw Open*. Published December 13, 2024.
doi:10.1001/jamanetworkopen.2024.50635

Data
Additional Information: Name of the trial registry：Intervention Study on Postpartum Contraceptive Service Registry's URL：https://www.chictr.org.cn/showproj.html?proj=56283
Trial registration number：ChiCTR2000034603
Data available: Yes
Data types: Deidentified participant data
How to access data: h_jiang@fudan.edu.cn
When available: With publication

Supporting Documents
Document types: None

Additional Information
Who can access the data: Researchers whose proposed use of the data has been approved
Types of analyses: For the purpose of repeating the current analysis
Mechanisms of data availability: Data will be made available after approval of a proposal and with a signed data access agreement.
